# Standardization of Health Terminology Systems and the Roles of Responsible Organizations

**Published:** 2018-10

**Authors:** Farid KHORRAMI, Maryam AHMADI, Abbas SHEIKHTAHERI

**Affiliations:** Dept. of Health Information Management, School of Health Management and Information Sciences, Iran University of Medical Sciences, Tehran, Iran

## Dear Editor-in-Chief

A terminology system is a set of words that represent a variety of concepts in a specific domain. Terminology systems may have many forms, such as nomenclatures, classifications, ontologies, lists, definitions, and any word sets used to record concepts (1). Today, health terminology systems are constantly evolving to address the dynamic challenges of computerizing clinical data and speeding up the implementation of electronic health records. Therefore, classification systems for diseases or medical interventions such as the 10th revision of the International Statistical Classification of Diseases and Related Health Problems (ICD-10), Current Procedural Terminology (CPT), or International Classification of Diseases, Ninth Revision, Clinical Modification (ICD-9-CM) cannot be used alone for the documenting the details of clinical records and making these data re-usable for secondary uses such as supporting disease registry systems, conducting medical researches and also quality management programs (1, 2). Researchers have pointed out this issue in several studies (3–6). Therefore, the strategic role of terminology systems as the interface for knowledge sources such as clinical guidelines, reminders, clinical decision support systems (CDSS), as well as support for practical analysis of quality improvement, clinical epidemiology and outcome analysis, should be given further consideration (2).

In general, there are two approaches to using health terminology systems:

- Using international or national terminologies of other countries,
- The creation of a new terminology based on the needs of the country (7).

Certainly, for the use of either of these two approaches, there should be appropriate criteria for choosing the terminology systems. In most countries, an organization, office or a standardization committee is responsible for solving this problem (8, 9). Therefore, regarding the standardization of terminology systems in the country, several main questions can be investigated:

1. Is an appropriate steward organization assigned for the several tasks usually undertaken by the standardization organizations in the field of health terminologies?
2. Is the governance authorities required to standardize the health terminologies for different fields such as medicine, public health, clinical findings, etc. assigned to the relevant organization?
3. Are there any clear and well-defined criteria for assessing and selecting a comprehensive terminology system in the country?
4. Are standard terminology systems identified for software vendors and other target groups?
5. Is there a mechanism for accessing software vendors and other target groups to the selected terminology systems?
6. Is there a well-defined mechanism for using terminology systems for multiple purposes, such as generating clinical guidelines or developing CDSS software?

The above-mentioned and many other questions addressed in the field of standardization of terminology systems have the potential to be a part of the research priorities of the country and the results can be applied in making policies in the area of standardization of electronic health records and the other forms of electronic documentation of clinical data. Indeed, without recording structured data with using standardized terminology systems and complying with international and national standards, we cannot expect to achieve the intended goals of using and re-using of electronic data captured in health records but using standalone systems.
